# Analysis of 915 MHz LoRa Technology in Underwater Environments: Feasibility and Applications in Aquatic Monitoring

**DOI:** 10.3390/s26154809

**Published:** 2026-07-29

**Authors:** Diego Montezuma-Rosero, Fabián Cuzme-Rodríguez, Jaime Michilena-Calderón, Luis Suárez-Zambrano, Carlos Vásquez-Ayala

**Affiliations:** 1 Facultad de Ingeniería en Ciencias Aplicadas, Universidad Técnica del Norte, Av. 17 de Julio 5-21 y General José María Córdova, Ibarra 100105, Ecuador; damontezumar@utn.edu.ec (D.M.-R.); jrmichilena@utn.edu.ec (J.M.-C.); lesuarez@utn.edu.ec (L.S.-Z.); cavasquez@utn.edu.ec (C.V.-A.); 2 Escuela Técnica Superior de Ingeniería de Telecomunicación, Universidad de Málaga, 29071 Málaga, Spain

**Keywords:** LoRa, underwater communications, wireless sensor networks, data communication, electromagnetic wave attenuation

## Abstract

Electromagnetic underwater communications are strongly limited by water conductivity and depth, particularly at ISM frequencies above 900 MHz. Although LoRa technology has been widely adopted in low-power wireless sensor networks, experimental studies of LoRa-based underwater-to-overwater (UW2OW) links at 915 MHz in real freshwater environments remain scarce. This work presents an experimental evaluation of a 915 MHz LoRa UW2OW communication link conducted in three freshwater scenarios with different conductivity conditions: a controlled swimming pool and two natural lakes. The experimental campaign analyzes the influence of water depth and horizontal distance on key performance metrics, including received signal strength indicator (RSSI), signal-to-noise ratio (SNR), packet loss rate, and end-to-end latency. Measurements were carried out at depths between 0.25 m and 0.8 m. Multiple spreading factors were evaluated in the pool scenario, while SF12 was selected for the natural-lake experiments. The results demonstrate that short-range UW2OW communication is feasible, achieving effective distances of up to 13 m at shallow depths. However, increased depth and higher water conductivity lead to significant performance degradation, with packet loss rates exceeding 80% at longer distances. The obtained results provide empirical insights and practical design criteria for short-range freshwater underwater wireless sensor networks based on LoRa technology.

## 1. Introduction

Underwater wireless sensor networks (UWSNs) are increasingly used in applications such as environmental monitoring, limnological studies, aquaculture, and the inspection of aquatic infrastructures. In these scenarios, submerged Sensor Nodes are required to transmit data reliably to surface gateways or remote monitoring stations, often under strict energy and hardware constraints. Achieving stable underwater communication under these conditions remains a challenging engineering problem.

Acoustic communication is currently the most established solution for underwater links, mainly due to its long achievable transmission ranges. However, acoustic systems are inherently affected by high propagation delays, limited data rates, and sensitivity to environmental noise and multipath effects, which restrict their use in latency-sensitive or low-power sensor applications [1,2]. Optical communication, on the other hand, can provide high data rates but is strongly limited by absorption, scattering, and water turbidity, making it suitable only for very short distances and highly controlled environments [3]. For short-range and low-latency applications, electromagnetic (EM) communication has therefore emerged as a complementary alternative, particularly when direct underwater-to-overwater (UW2OW) links are required [4].

In practice, EM wave propagation in water is strongly influenced by frequency-dependent attenuation, water conductivity, and sensor deployment depth. These factors severely limit communication range, especially at frequencies above several hundred megahertz. Nevertheless, the availability of unlicensed ISM frequency bands enables the use of low-cost and low-power radio technologies that are attractive for sensor-based underwater monitoring systems.

LoRa technology has been widely adopted in terrestrial low-power wide-area networks due to its robustness, long communication range, and low energy consumption. In recent years, several studies have explored the feasibility of LoRa-based underwater communication, mostly operating at 433 MHz and 868 MHz and often under controlled laboratory or pool conditions [5,6,7,8,9]. While these studies demonstrate the potential of LoRa for underwater sensing applications, experimental results obtained in real freshwater environments remain limited. In particular, the behavior of LoRa links operating at 915 MHz has not been sufficiently investigated, especially when water depth, horizontal distance, and conductivity vary simultaneously in practical deployments.

To address this gap, this study experimentally evaluates a 915 MHz LoRa underwater-to-overwater (UW2OW) communication link in three freshwater scenarios with distinct conductivity characteristics, namely a controlled swimming pool and two natural lakes. The analysis focuses on the combined effect of water depth, horizontal separation distance, and medium conductivity on received signal strength indicator (RSSI), signal-to-noise ratio measured at the receiver (SNR), packet loss rate, and end-to-end latency.

The contribution of this work should be interpreted as an extension of an already established line of research on underwater electromagnetic and LoRa-based communications rather than as evidence of a fundamentally different propagation mechanism between the 868 MHz and 915 MHz bands. In this sense, the novelty lies primarily in the experimental characterization of the 915 MHz LoRa band used in ITU Region 2 and in the comparative evaluation of real freshwater environments with different conductivity conditions.

The main contributions of this work can be summarized as follows:

1. An experimental characterization of a 915 MHz LoRa UW2OW communication link in multiple freshwater environments with different conductivity levels.
2. A comparative analysis of the effect of node depth and horizontal distance on RSSI, SNR, packet loss, and latency under practical deployment conditions.
3. An empirically grounded operating envelope for short-range freshwater monitoring applications based on the tested LoRa platform and deployment configuration.

Accordingly, this paper does not claim the design of a new antenna, coding scheme, or full propagation model. Instead, it provides a reproducible experimental characterization of the tested communication system and discusses the main physical and implementation factors that constrain performance in shallow freshwater environments.

The remainder of this paper is organized as follows. Section 2 reviews related work on electromagnetic and LoRa-based underwater communication systems. Section 3 describes the experimental setup and methodology. Section 4 presents the experimental results, and Section 5 discusses the implications of the findings. Finally, Section 6 concludes the paper and outlines future research directions.

## 2. Related Work

Studies focused on electromagnetic underwater communication have explored different frequencies and deployment configurations. In [7], a LoRa-based Sensor Node operating at 433 MHz was evaluated using spreading factor (SF) 12 and 125 kHz bandwidth under UW2UW, UW2OW and OW2UW configurations. The results showed that links with only one submerged node provided more stable communication compared to fully submerged setups.

A comparative evaluation between LoRaWAN and NB-IoT for underwater-to-above-water communication was presented in [8]. Using a multiprotocol prototype, the authors reported attenuation values close to 30 dB for LoRaWAN in underwater scenarios. In addition, the use of RF electromagnetic signals in underwater sensor networks was analyzed in [9], highlighting that although acoustic systems provide longer ranges, electromagnetic signals offer the advantage of crossing the water–air interface more easily, which is relevant when surface buoys are used.

Other works have evaluated LoRa technology at 868 MHz. In [10], a LoRaWAN Sensor Node enclosed in an IP68 box was tested in a swimming pool, reaching a maximum submersion depth of approximately 1.10 m when operating at SF12. Beyond LoRa, different antenna and radio approaches have also been investigated. For example, Wi-Fi communication at 2.4 GHz has been reported to achieve only very short underwater propagation distances, on the order of 0.15 m [11], whereas a dipole antenna operating at 38 MHz reached up to 5.5 m at 10 dBm transmission power [12]. Low-frequency experiments based on BPSK modulation at 3 kHz have also been reported, obtaining communication ranges of up to 40 m between submerged nodes [11].

Propagation studies such as [13] analyzed electromagnetic wave behavior in shallow seawater using loop antennas placed on the seabed, with test distances between 2 m and 6 m. The results indicated that frequencies between 10 kHz and 100 kHz are less affected by the medium compared to higher frequencies. Additionally, Ref. [14] presented the development of an underwater antenna designed for LoRa communication at 868 MHz, achieving transmission distances up to 6 m, with received signal levels around −110 dBm before link loss.

Although these studies demonstrate the feasibility of electromagnetic and LoRa-based underwater communication, most experiments were performed at 433 MHz or 868 MHz and often under controlled conditions. Experimental evaluations at 915 MHz in natural freshwater environments remain limited, particularly when depth and horizontal distance are varied simultaneously.

## 3. Materials and Methods

### 3.1. Hardware Setup

The experimental platform was based on the Heltec Wi-Fi LoRa 32 V3 development board (Heltec Automation, Chengdu, China), which integrates an ESP32-S3 microcontroller (Espressif Systems, Shanghai, China) and an SX1262 sub-GHz LoRa transceiver (Semtech Corporation, Camarillo, CA, USA), allowing firmware development through the Arduino IDE version 2.3.6 environment [15,16]. Since the purpose of this work is not to compare commercial modules but to characterize underwater communication using a reproducible low-cost platform, only the main technical specifications required to understand the communication setup are summarized in Table 1 [15].

### 3.2. System Architecture

The proposed system comprises a submerged Sensor Node, a surface Communication Buoy, and a backend for data storage and visualization. From the communication-analysis perspective, the critical link is the 915 MHz LoRa UW2OW transmission between the submerged node and the buoy. The backend is included only to support data collection and monitoring, but it is not the main object of the radio-link analysis presented in this paper.

**Figure 1:**
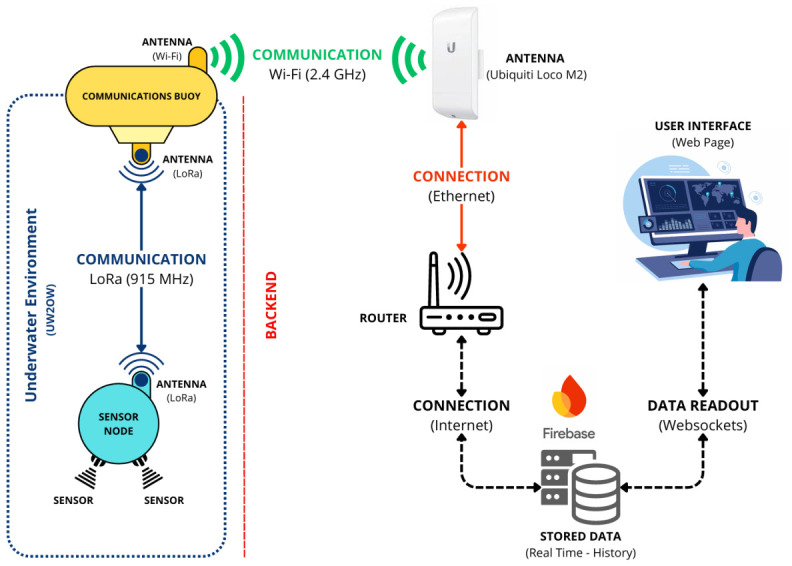
System architecture illustrating the communication process between the Sensor Node and the Communications Buoy.

The system architecture shown in Figure 1 illustrates the complete operational process of the proposed structure, showing how its components interact to evaluate LoRa communication in the underwater environment. The Sensor Node acquires pH and turbidity data and transmits them through the 915 MHz LoRa channel. The Communication Buoy receives the packet at the water surface and stores the corresponding reception parameters for later analysis. In the complete system, the buoy also retransmits the received information through Wi-Fi using a NanoStation Loco M2 access point (Ubiquiti Inc., New York, NY, USA) to a remote backend hosted on Firebase (Google LLC, Mountain View, CA, USA). However, the performance metrics analyzed in this paper are associated with the UW2OW LoRa link.

### 3.3. Communication Parameters

The communication parameters were selected to maximize reception robustness in a highly attenuating underwater medium while preserving a reproducible low-cost configuration. Since the experiments were conducted in ITU Region 2, the operating frequency was set to 915 MHz, within the 902–928 MHz ISM band supported by the Heltec Wi-Fi LoRa 32 V3 module [15,17]. The transmission power was fixed at 21 dBm in order to maximize signal coverage under underwater conditions, despite the corresponding increase in power consumption [17,18].

Bandwidth was fixed at 125 kHz because it provides a suitable compromise between data rate and receiver sensitivity for low-power LoRa links [15,17]. The spreading factor (SF) was treated as the main robustness–throughput control parameter. Higher SF values increase receiver sensitivity and improve tolerance to weak signals, at the cost of lower data rate [17,19]. For this reason, SF values from SF7 to SF12 were evaluated in the pool scenario, whereas SF12 was selected for the lake experiments because it provided the highest communication margin during the preliminary tests. The coding rate (CR) was fixed at 4/8 to prioritize redundancy and link robustness during the underwater measurements [19,20].

To support the later discussion of the throughput–reliability trade-off, the bitrate $R_{b}$ was estimated as [19]:

(1) $$R_{b}=SF\cdot \frac{BW}{2^{SF}}\cdot CR,$$

where BW is the bandwidth, SF is the spreading factor, and CR is the coding rate. Under the adopted configuration (BW=125 kHz and CR=4/8), the estimated bitrate decreases from 3417.7 bps at SF7 to 183.12 bps at SF12, illustrating the expected robustness–throughput trade-off.

In addition, the packet was configured using the explicit header mode, with CRC enabled, a preamble length of 12 symbols, and an application payload of approximately 58 bytes. Since the payload had to remain within the configured transmission buffer, the buffer size was set to 60 bytes [16]. Table 2 summarizes the parameter configuration used during the underwater tests.

### 3.4. LoRa Packet Format

**Figure 2:**
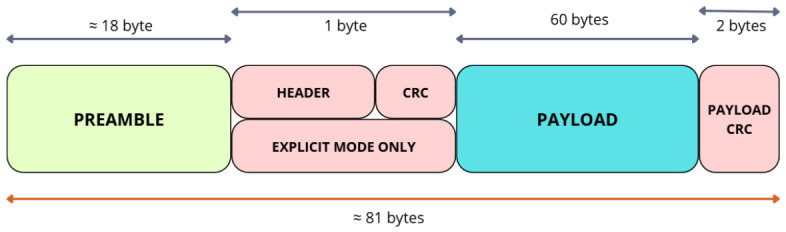
Configuration of each of the parts that make up the LoRa packet.

The experiments used the LoRa explicit packet mode with CRC enabled and a preamble of 12 symbols. Under this configuration, the final packet size was 81 bytes, as illustrated in Figure 2.

### 3.5. Test Devices

The devices used to perform the underwater communication tests are based on an Underwater Node, which contains a Heltec Wi-Fi LoRa 32 V3 module (Heltec Automation, Chengdu, China). This module collects data from the bottom of the water, such as pH and turbidity, through connected sensors. The data are transmitted using an omnidirectional communication antenna encapsulated inside an Acrylonitrile Butadiene Styrene (ABS) enclosure. ABS is a material widely used in the manufacture of housings for electronic devices due to its flexibility and impact resistance [21]. It has a relative permittivity ($\epsilon_{3}$) of 2.5 [22].

**Figure 3:**
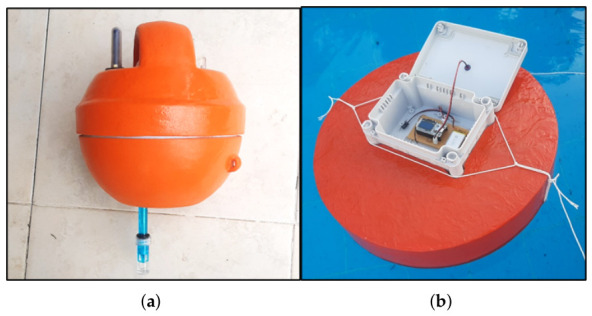
Underwater Node (**a**). Communication Buoy (**b**).

It should be noted that both the antenna in the Underwater Node and the antenna in the Receiving Buoy use the same encapsulation process. This configuration requires a small air interface that allows the electromagnetic wave to be emitted and propagate into the aquatic environment [23]. Figure 3a shows the Underwater Node used in this study, while Figure 3b presents the Receiving Buoy.

Both the Underwater Node and the Receiving Buoy used the integrated PCB antenna provided by the Heltec Wi-Fi LoRa 32 V3 module, operating at 915 MHz with an approximate antenna gain of 2 dBi. The antenna was vertically oriented during all experiments in order to maintain polarization consistency between the transmitter and receiver.

The communication modules were enclosed inside cylindrical ABS enclosures with an approximate diameter of 13 mm, a height of 55 mm, and an estimated wall thickness of 1.5 mm. The antenna was positioned close to the upper internal section of the enclosure, leaving a small internal air gap between the antenna and the ABS wall. During the tests, the antenna of the Underwater Node remained completely submerged, while the antenna of the Receiving Buoy was positioned near the air–water interface.

Both devices were powered using rechargeable 3.7 V lithium batteries. The firmware was developed in Arduino IDE using the LoRaWan_APP.h library version 2.0.0 for the SX1262 LoRa transceiver with the explicit header mode, CRC enabled, bandwidth of 125 kHz, coding rate 4/8, and SF configurations ranging from SF7 to SF12 depending on the experiment as summarized in Table 3.

### 3.6. Test Environments

It is important to clarify how the separation distance between the nodes was measured in the test environments. The distance was measured horizontally along the water surface, that is, from the point where the Sensor Node is submerged to the location of the Communications Buoy.

**Figure 4:**
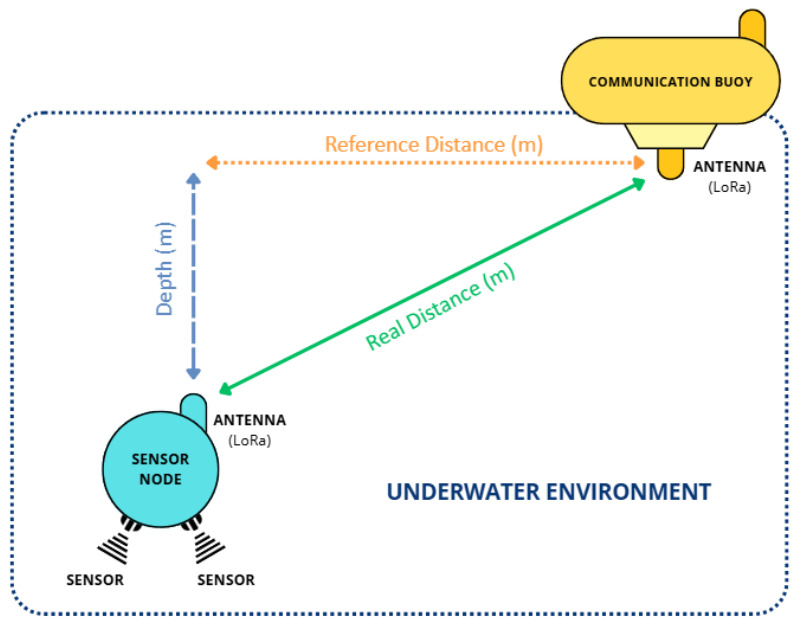
Distance measurement method.

Therefore, the distances shown in the graphs correspond to this reference measurement. However, the actual separation distance between the nodes can be calculated using the Pythagorean theorem, since it represents the diagonal distance between the antenna of the Underwater Node and the antenna of the Communications Buoy, as illustrated in Figure 4.

**Figure 5:**
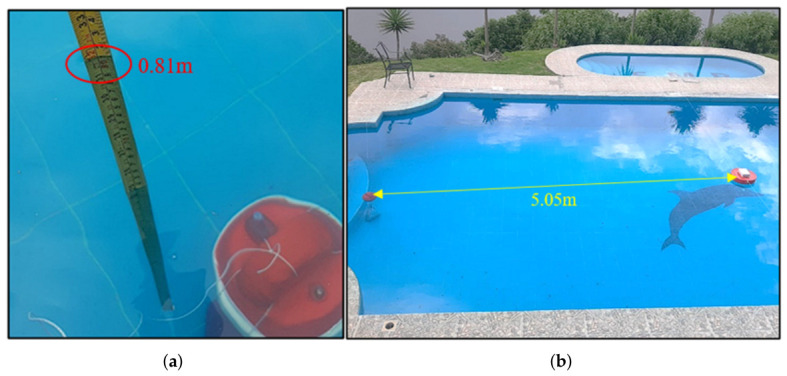
Pool test scenario: experimental setup showing the Underwater Node depth (**a**) and the node separation distance (**b**).

One of the initial communication test environments of the system is a swimming pool, where the node was placed at a depth of 0.81 m, as shown in Figure 5a. Figure 5b illustrates how the separation distance was measured, starting from the point where the Underwater Node is submerged to the location of the Receiving Buoy.

**Figure 6:**
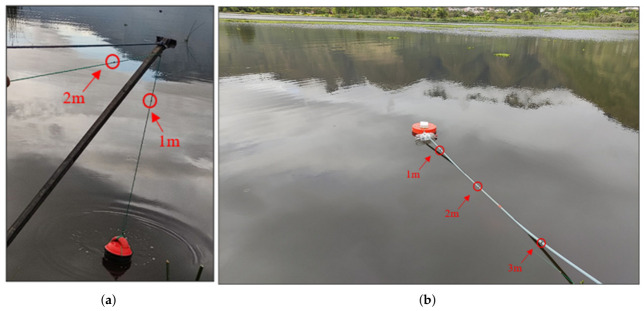
Lagoon test environment (Yahuarcocha): pulley system used to submerge the Underwater Node (**a**) and positioning of the Receiving Buoy at different distances (**b**).

Another scenario in which the system was evaluated is the Yahuarcocha Lagoon, located in the city of Ibarra (Ecuador). A pulley system with a rope marked at one-meter intervals was used to provide a reference for the depth at which the Underwater Node was submerged, as shown in Figure 6a.

Similarly, a support tube marked at one-meter intervals was used to measure the horizontal separation distance on the water surface between the Receiving Buoy and the point where the Underwater Node was submerged, as illustrated in Figure 6b. The node was submerged at depths of 0.25 m, 0.5 m, and 0.8 m.

**Figure 7:**
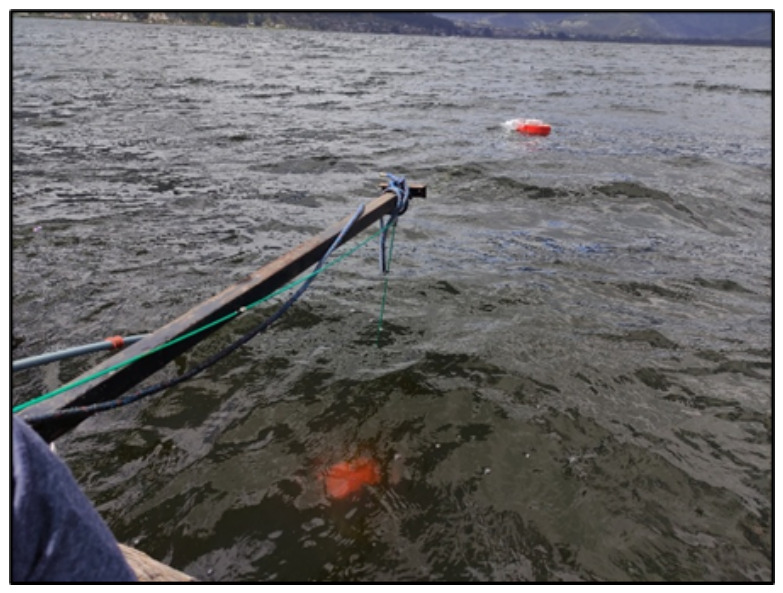
Field setup used for the communication tests in San Pablo Lagoon.

The final scenario in which the system communication tests were performed was the San Pablo Lagoon, located in the province of Imbabura (Ecuador). The same methodology used in the Yahuarcocha Lagoon was applied in this case. This test environment is shown in Figure 7. The node was submerged at depths of 0.25 m, 0.5 m, and 0.8 m.

The test environments corresponding to the two lagoons were selected due to the distinct characteristics of each medium, particularly the conductivity values in both underwater environments. A comparison of these characteristics is presented in Table 4.

## 4. Results

### 4.1. Pool Test Environment

The pool tests were conducted with the Sensor Node submerged at a depth of 0.8 m. The reference separation distance between both nodes was evaluated from 0.3 m to 11.4 m. This procedure was performed for each of the available spreading factor (SF) values in LoRa. LoRa modulation can tolerate SNR values as low as approximately −20 dB when operating with SF12. Below this threshold, the signal becomes too corrupted to be properly detected.

In this paper, SNR denotes the signal-to-noise ratio reported by the LoRa receiver during packet reception. The communication limit depends on the selected spreading factor because the receiver sensitivity threshold changes with SF. Under the tested configuration, high spreading factors such as SF12 preserve communication at substantially lower SNR values than low spreading factors such as SF7.

**Figure 8:**
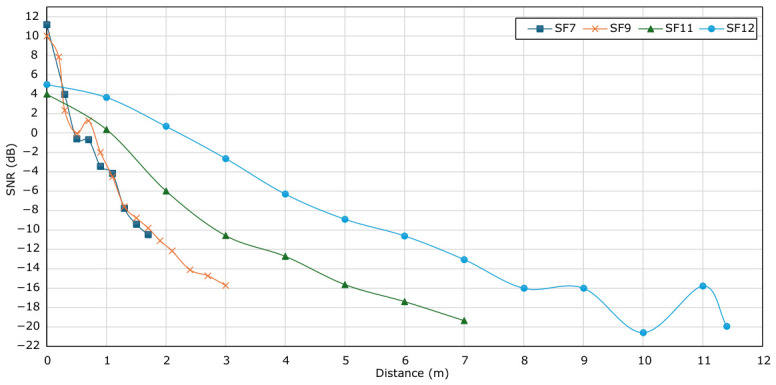
SNR as a function of horizontal distance for different spreading factors in the pool test environment.

Figure 8 presents the evaluated signals. The results show that the configuration using SF12 achieved the longest communication range, reaching up to 11.4 m before the signal was completely lost. At a distance of 10 m, the SNR value is already close to −20 dB, indicating that at 11.4 m the signal is received with significant difficulty.

The abrupt initial drop observed for SF7 and SF9 in Figure 8 can be associated with the combined effect of lower receiver sensitivity and the radiation pattern of the integrated omnidirectional PCB antenna under vertical orientation. In the near-field and short-range submerged configuration, the relative alignment between the antenna lobes and the underwater-to-overwater path may produce an initially unfavorable coupling condition, especially for low-SF configurations with reduced sensitivity margin. As the submerged node is displaced horizontally, the effective propagation geometry becomes more stable, and the attenuation trend evolves toward the expected distance-dependent decay in the lossy medium.

In contrast, SF7, which is the lowest configurable value, achieved the shortest transmission distance, reaching only 1.7 m. This represents nearly a 9 m reduction compared to the SF12 configuration. The SNR decay for SF11 and SF12 remains relatively gradual up to approximately 2.1 m. Beyond this distance, the degradation becomes more pronounced as the separation distance increases, particularly for SF11. Conversely, lower spreading factors such as SF7 exhibit a faster SNR degradation from shorter distances, leading to earlier communication loss (around 0.5 m under similar conditions).

It is important to note that the minimum tolerable SNR threshold in LoRa depends on the configured spreading factor. For example, when using SF7, communication is typically lost at approximately −10 dB, whereas higher spreading factors such as SF12 can operate at SNR values close to −20 dB.

Parameters such as received signal strength (RSSI) and delay were also evaluated for each spreading factor (SF) value. Table 5 presents the average results obtained during the tests. In the case of RSSI, all signals reached approximately −116 dBm before being lost, regardless of the configured SF value.

On the other hand, the delay is lower for signals configured with SF7, with an average value of approximately 409 ms. However, this value increases to 5940 ms when using an SF12 configuration, confirming that a higher SF increases transmission time and overall delay. Similarly, the minimum SNR values reached before signal loss are observed for each SF, with SF7 showing the lowest tolerance.

### 4.2. Yahuarcocha Lagoon Test Environment

The tests conducted in the Yahuarcocha Lagoon produced results different from those obtained in the swimming pool environment. In this case, only the signal configured with SF12 was evaluated, as it was previously identified as the configuration providing the greatest communication range. The tests were performed with the Sensor Node submerged at different depths: 0.25 m, 0.5 m, and 0.8 m.

**Figure 9:**
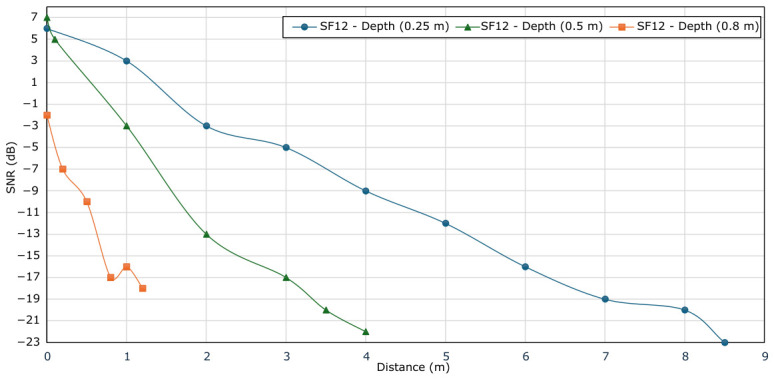
SNR versus distance at different depths using SF12 in the Yahuarcocha Lagoon test environment.

The experimental results indicate that at a depth of 0.25 m, the signal propagates up to a distance of 8.5 m. However, as the depth of the Sensor Node increases, the communication range decreases. As shown in Figure 9, the signal evaluated at a depth of 0.5 m reaches a maximum distance of 4 m, while the signal at 0.8 m depth is the most affected, reaching only 1.2 m.

**Figure 10:**
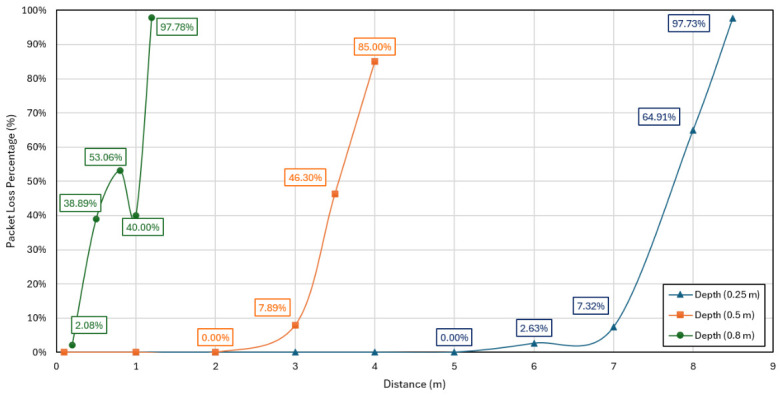
Packet loss graph at different depths using SF12 in the Yahuarcocha Lagoon test environment.

In accordance with the above results, Figure 10 presents the packet loss percentage for each signal evaluated at the three depths. The results indicate that the signal evaluated at a depth of 0.25 m begins to experience packet loss of 2.63% at a distance of 6 m. This percentage increases to 64.91% at 8 m and rises to nearly 100% at 8.5 m. Therefore, although the maximum communication range reaches 8.5 m, the optimal operating distance is realistically between 7 m and 8 m, considering the packet loss rate.

The signal evaluated at a depth of 0.5 m begins to show losses at 3 m, with 7.89% packet loss, increasing to nearly 85% at 4 m. Finally, the signal evaluated at a depth of 0.8 m exhibits the highest losses. Packet loss begins at 0.2 m and gradually increases up to 0.8 m, reaching 53%. An improvement is observed at 1 m, where packet loss decreases to 12%. However, when the Communications Buoy is moved slightly further to a distance of 1.2 m, the packet loss increases to 97% before the signal is completely lost.

A table presenting the obtained results is shown, where the maximum distances reached at each evaluated depth are displayed. It is noteworthy that the RSSI values remained constant at −116 dBm as the maximum value before signal loss. In the case of SNR, the minimum value reached was −23 dB for the signal evaluated at a depth of 0.25 m, which is below the approximate −20 dB LoRa reception threshold. However, this value must be interpreted together with the nearly 100% packet loss observed when the signal reached 8.5 m.

The highest delay was observed for the signal evaluated at a depth of 0.25 m, while the lowest delay corresponded to the intermediate depth of 0.5 m. Table 6 summarizes the data obtained from this test conducted in Yahuarcocha Lagoon.

### 4.3. San Pablo Lagoon Test Environment

**Figure 11:**
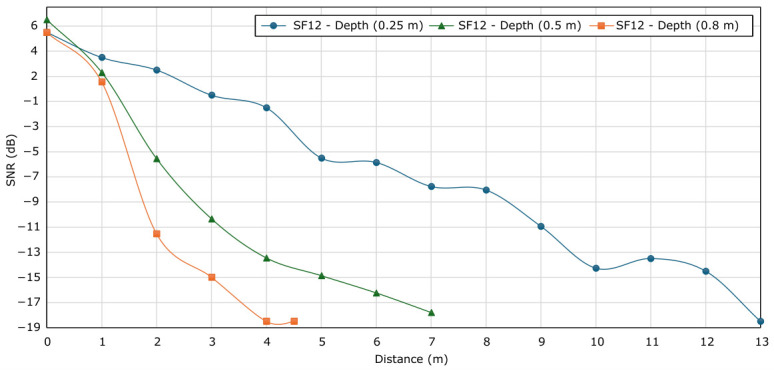
SNR versus distance at different depths using SF12 in the San Pablo lagoon test environment.

Regarding the tests conducted in San Pablo Lagoon, the same experimental conditions used in Yahuarcocha Lagoon were applied, namely three different depths (0.25 m, 0.5 m, and 0.8 m). The results are presented in Figure 11, where it can be observed that the three signals initially exhibit values close to 6 dB. However, the signal measured at a depth of 0.25 m shows a more gradual decrease per meter of distance between nodes, reaching −18 dB before being completely lost at 13 m.

In contrast, the signal measured at 0.8 m depth exhibits the fastest degradation in its SNR parameter as the distance increases. At approximately 4 m, it already reaches −18 dB and remains at this level until it attains its maximum distance of 4.5 m, at which point the signal is completely lost.

**Figure 12:**
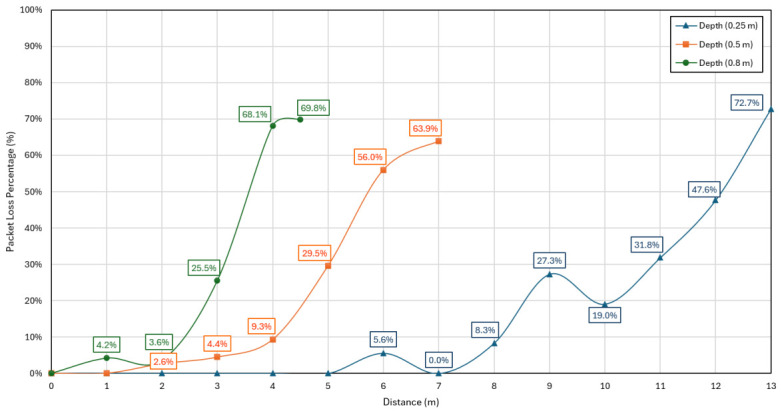
Packet loss graph at different depths using SF12 in the San Pablo lagoon test environment.

Figure 12 presents the packet loss results for each signal evaluated at the three depths (0.25 m, 0.5 m, and 0.8 m). For the 0.8 m depth, packet loss begins at 1 m with a value of 4.17%, increasing to 25.49% at 3 m. Beyond this distance, the loss becomes significant, reaching nearly 70% at 4.5 m.

For the signal evaluated at 0.5 m depth, packet loss begins at 2 m with 2.63% and remains at relatively low values up to 5 m, where it increases to approximately 30%. This value rises to 56% at 6 m and reaches a maximum of 63.89% at 7 m, just before complete signal loss.

Finally, the signal evaluated at 0.25 m depth exhibits a noticeable packet loss at 9 m, reaching approximately 27%. A slight improvement is observed at 10 m (19% loss); however, the loss increases again at 11 m to 31.8% and rises sharply to 72.73% at 13 m, where the signal is completely lost.

The values summarized in Table 7 indicate that the maximum communication distance was achieved at 13 m for the 0.25 m depth, whereas the shortest distance was 4.5 m at 0.8 m depth. The RSSI values remained constant at −116 dBm prior to complete signal loss, while the SNR values were above the −20 dB tolerance threshold specified for LoRa.

This test also exhibited the highest delay values, reaching 6403 ms for the signal evaluated at 0.25 m depth, which corresponded to the longest transmission time.

### 4.4. General Results

**Figure 13:**
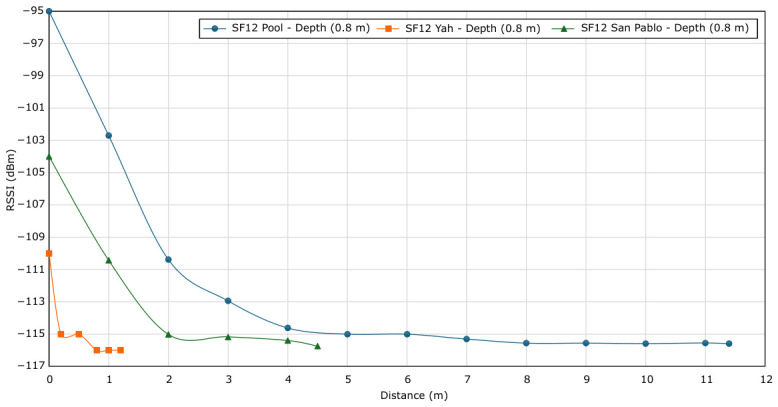
RSSI at 0.8 m depth: comparison across the three test scenarios.

Figure 13 compares the RSSI values obtained in the three test scenarios at a depth of 0.8 m. Under this common depth condition, the longest communication range was achieved in the pool environment (11.4 m), followed by San Pablo Lagoon (4.5 m), whereas Yahuarcocha Lagoon showed the shortest range (1.2 m). These results indicate that the propagation medium strongly affects LoRa performance under UW2OW conditions.

**Figure 14:**
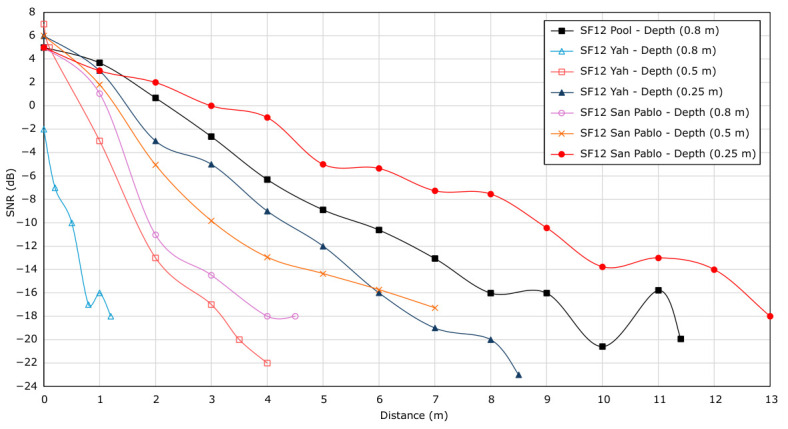
SNR comparison using SF12 configuration for all evaluated signals.

A similar trend is observed in the SNR comparison shown in Figure 14. At 0.8 m depth, the Yahuarcocha signal experiences the fastest degradation, reaching values close to the communication limit at much shorter distances than in the other two scenarios. In contrast, the pool environment preserves a more gradual decay, while San Pablo Lagoon exhibits intermediate behavior. This comparative result is consistent with the conductivity differences reported for both natural lakes.

Table 8 summarizes the main comparative results for the three scenarios at 0.8 m depth. For all cases, the RSSI values remained close to −116 dBm before complete signal loss, while the limiting SNR values were close to the expected LoRa reception threshold for SF12. Regarding delay, the lowest value was observed in Yahuarcocha Lagoon, whereas the highest delay was recorded in San Pablo Lagoon.

Overall, the results confirm that both the characteristics of the propagation medium and the submersion depth play a critical role in determining the achievable communication range under freshwater UW2OW operation.

## 5. Discussion

The obtained results confirm that LoRa-based UW2OW communication at 915 MHz is feasible in shallow freshwater environments but strongly constrained by the combined effect of conductivity, node depth, and receiver sensitivity. The measured trends are consistent with the expected behavior of electromagnetic propagation in lossy media. As the Sensor Node is placed deeper, a larger fraction of the propagation path remains inside water before the signal reaches the air–water interface, thereby increasing attenuation. This explains why the best performance was systematically observed at 0.25 m depth, whereas the shortest communication ranges occurred at 0.8 m depth.

Considering the work reported in [7], which evaluated UW2OW, OW2UW, and UW2UW configurations using similar LoRa parameters (SF12, 125 kHz bandwidth, 20 dBm transmission power, coding rate 4/5, and a frequency of 433 MHz), it was observed that configurations in which both the transmitter and receiver were submerged exhibited earlier signal degradation. In contrast, the best performance was achieved when only one node was submerged and the other remained above the water surface (UW2OW configuration). This finding supports the configuration adopted in the present study.

The obtained results demonstrate that water conductivity significantly affects the propagation of LoRa signals at 915 MHz in freshwater environments. According to Table 4, Yahuarcocha Lagoon presents a conductivity of approximately 560 μS/cm, whereas San Pablo Lagoon exhibits lower values in the range of 300–320 μS/cm. Under comparable depth conditions, this difference is consistent with the shorter communication ranges observed in Yahuarcocha Lagoon and the longer ranges measured in San Pablo Lagoon.

The observed attenuation behavior can be explained by the conductive properties of freshwater, which introduce significant dielectric losses at 915 MHz. As conductivity increases, the effective propagation constant also increases, leading to stronger attenuation of the electromagnetic wave inside the medium. Consequently, even moderate differences in conductivity between freshwater bodies can produce substantial variations in the practical UW2OW communication range.

Furthermore, consistent improvements were observed at other evaluated depths in San Pablo Lagoon, reaching a maximum horizontal distance of 13 m at a depth of 0.25 m from the Sensor Node. Table 9 presents a comparison between the results obtained in this study and those reported in related works.

The obtained results show that the achieved depths are comparable to those reported in previous studies, ranging between 0.8 m and 1.25 m depending on the operating frequency and node configuration. However, the main contribution of this work lies in the evaluation of three distinct aquatic scenarios with different environmental characteristics, as well as the assessment of a higher operating frequency (915 MHz).

In addition to comparisons with similar LoRa-based technologies, it is also relevant to contrast the results with other communication technologies applied in underwater environments. For this purpose, Table 10 presents a comparative analysis of different approaches reported in the literature.

Operating frequency is a critical parameter influencing underwater communication range. For example, at a frequency of 38 MHz, transmission depths of up to 2.5 m have been reported in [11], exceeding the depths achieved in the present study. In contrast, at higher frequencies such as 2.4 GHz, the propagation distance is significantly reduced, typically to only a few centimeters due to increased attenuation.

The present work evaluates LoRa signal propagation across three distinct aquatic scenarios and at multiple depths. Although maximum communication distances were obtained under specific conditions, these values do not necessarily represent optimal operating ranges for reliable data transmission. Therefore, Table 11 presents the recommended distances for stable data exchange between a Sensor Node and a communication buoy under the UW2OW configuration.

At the recommended operating distances, packet loss remains below 50%. This level should not be interpreted as a general reliability target but rather as a practical upper bound for the tested low-rate monitoring scenario, where periodic sensing, temporal redundancy, and application-layer tolerance may still allow useful data recovery. Therefore, the distances reported in Table 11 should be interpreted as empirical operating points for the evaluated platform and deployment conditions.

The observed attenuation behavior is strongly influenced by the conductive properties of freshwater, which introduce significant dielectric losses at 915 MHz. Spatial variations in conductivity, influenced by dissolved ions and sediment interactions, increase the effective propagation constant and result in higher signal attenuation.

These results confirm the dominant role of medium conductivity: under comparable depth conditions, San Pablo Lagoon achieved longer communication distances than Yahuarcocha Lagoon, consistent with its lower reported conductivity.

From a system-design perspective, the SF12 configuration provided the largest communication margin but also the lowest bitrate. This trade-off is expected in LoRa systems. Using the same bitrate relationship adopted in this paper, a moderate increase in throughput can be obtained by reducing redundancy in the coding rate while preserving the same bandwidth and spreading factor. For example, changing from CR=4/8 to CR=4/7 yields an increase of approximately 14.3%, whereas changing from CR=4/8 to CR=4/6 yields an increase of approximately 33.3%, at the cost of reduced error-correction capability. Additional marginal gains may also be achieved through payload optimization, shorter preamble settings in stable channels, or the use of lower spreading factors in shallower and lower-loss conditions. Accordingly, the practical operating point must be selected as a compromise between communication robustness and a useful data rate.

The field measurements in the natural lakes were performed under real surface conditions, where visible waves and local water motion may affect the received signal. Under such conditions, the air–water interface is not perfectly static, which may generate time-varying reflections and a dynamic multipath. Likewise, the supporting platform used during the field deployment may perturb the local electromagnetic environment depending on its material composition and geometry. These effects may influence local fluctuations and short-range irregularities in the measured data. However, they do not invalidate the broader trends observed across depth and conductivity conditions because the same experimental methodology was maintained within each scenario and the differences between scenarios remained systematic.

The comparison with previous studies must also be interpreted carefully. The present work does not claim a universal performance limit for LoRa underwater communication. Instead, it provides an experimentally grounded characterization of one specific platform, namely the Heltec Wi-Fi LoRa 32 V3 with SX1262 transceiver, integrated PCB antenna, and the tested enclosure strategy. Different LoRa modules may exhibit variations in PCB layout, antenna efficiency, receiver noise figure, and frequency accuracy. Therefore, the absolute values reported here should be interpreted as platform-dependent results obtained under the tested UW2OW configuration.

The main limitations of this study are the use of a single hardware platform, shallow freshwater conditions, static deployment geometry, and the absence of dedicated multi-platform validation. Accordingly, future work should address multi-hardware comparison, dedicated antenna redesign, experiments under stronger dynamic surface conditions, and refined empirical or semi-empirical propagation models derived from larger datasets.

## 6. Conclusions

This study experimentally evaluated the feasibility of 915 MHz LoRa communication in underwater-to-overwater configurations for short-range freshwater monitoring scenarios. The results show that communication performance is mainly influenced by spreading factor, submersion depth, and medium conductivity.

Among the evaluated configurations, SF12 provided the highest robustness and the longest communication ranges. The best performance was consistently obtained at shallow depths, where a shorter portion of the propagation path remains inside water before the signal crosses the air–water interface. Likewise, lower-conductivity conditions, as observed in San Pablo Lagoon, resulted in longer communication distances than those measured in Yahuarcocha Lagoon. Under the tested conditions, the maximum communication distance reached 13 m in the UW2OW configuration.

The contribution of this work should be understood as an experimental characterization of the tested Heltec Wi-Fi LoRa 32 V3 / SX1262 platform and deployment configuration, not as a technology-wide limit for all LoRa implementations. Within this scope, the results provide useful empirical guidance for short-range aquatic monitoring systems requiring low-cost UW2OW data transmission.

The main limitations of this study are the use of a single hardware platform, shallow freshwater conditions, and static deployment geometry. Future work should therefore address multi-platform validation, improved antenna designs, measurements under dynamic surface conditions, and refined empirical or semi-empirical propagation models supported by larger datasets.

## Figures and Tables

**Table 1:** Technical data of Heltec Wi-Fi LoRa 32 V3 module.

Data	Information
Microcontroller	ESP32-S3
LoRa Chip	SX1262
Power Consumption	<10 µA
Frequencies	863–928 MHz, 470–510 MHz
Sensitivity	−134 dBm
Tx Power	±21 dBm
Modulation	FSK, CSS
Programmable Bitrate	62.5 Kbps LoRa and 300 kbps FSK
Communication Technologies	Bluetooth, Wi-Fi (802.11 b/g/n), LoRa
Operating Temperature	−20 °C to 70 °C
ROM	348 KB
SRAM	512 KB
RTC SRAM	16 KB
SiP Flash	8 MB
Battery Voltage	3.7 V

**Table 2:** Configured LoRa parameters.

Parameter	Value
Frequency	915 MHz
Power	21 dBm
Bandwidth	125 kHz
Spreading Factor	SF12
Coding Rate	4/8
Preamble Length	12 symbols
Buffer Size	60 bytes

**Table 3:** Additional hardware and antenna specifications.

Parameter	Description
Antenna Type	Integrated PCB antenna
Antenna Gain	Approximately 2 dBi
Polarization	Vertical
Encapsulation Material	ABS
Encapsulation Thickness	15 mm
Node Power Supply	3.7 V Li-ion battery
Operating Frequency	915 MHz

**Table 4:** Comparison of the characteristics of Yahuarcocha Lagoon and San Pablo Lagoon.

Characteristic	San Pablo Lagoon [24]	Yahuarcocha Lagoon [25]
Altitude above sea level	2662 m	2190 m
Maximum depth	35.2 m	6.5 m
Conductivity	300–320 µS/cm	560 µS/cm
Temperature	19 °C	11 °C

**Table 5:** Communication performance as a function of spreading factor.

Characteristic	SF7	SF9	SF11	SF12
Maximum referential data transfer distance	1.8 m	3 m	7 m	11.4 m
Maximum actual data transfer distance	1.93 m	3.08 m	7.04 m	11.42 m
Minimum RSSI reached	−115 dBm	−116 dBm	−116 dBm	−116 dBm
Minimum SNR reached	−11 dB	−17 dB	−20 dB	−20 dB
Average Delay	409 ms	535 ms	3416 ms	5940 ms

**Table 6:** Data obtained from tests conducted in Yahuarcocha Lagoon at three different depths.

Detail	Depth 0.25 m	Depth 0.5 m	Depth 0.8 m
Maximum referential data transfer distance	8.5 m	4 m	1.2 m
Maximum actual data transfer distance	8.5 m	4.01 m	1.4 m
Minimum RSSI reached	−116 dBm	−116 dBm	−116 dBm
Minimum SNR reached	−23 dB	−22 dB	−18 dB
Average Delay	5592 ms	5398 ms	5650 ms

**Table 7:** Data obtained from tests conducted in San Pablo Lagoon at three different depths.

Detail	Depth 0.25 m	Depth 0.5 m	Depth 0.8 m
Maximum referential data transfer distance	13 m	7 m	4.5 m
Maximum actual data transfer distance	13 m	7 m	4.55 m
Minimum RSSI reached	−116 dBm	−116 dBm	−116 dBm
Minimum SNR reached	−18 dB	−17 dB	−18 dB
Average Delay	6403 ms	6310 ms	6237 ms

**Table 8:** Comparative data from the three tests performed.

Detail	Pool SF12 Depth (0.8 m)	San Pablo SF12 Depth (0.8 m)	Yahuarcocha SF12 Depth (0.8 m)
Maximum referential data transfer distance	11.4 m	4.5 m	1.2 m
Maximum actual data transfer distance	11.42 m	4.55 m	1.39 m
Minimum RSSI reached	−116 dBm	−116 dBm	−116 dBm
Minimum SNR reached	−20 dB	−18 dB	−18 dB
Average Delay	5942 ms	6237 ms	5650 ms

**Table 9:** Comparison with other studies using similar technology.

Detail	Our Test Environment	Test Environment 1 [10]	Test Environment 2 [7]
Test environment	Pool, Yahuarcocha Lagoon, and San Pablo Lagoon	Pool	Dishui Lake
Technology	LoRa	LoRa	LoRa
Configuration	UW2OW	UW2AW	UW2OW
Depths evaluated	0.25 m, 0.5 m, and 0.8 m	0.2 m to 1.10 m	0.25 m to 1.75 m
Maximum data transfer distance	13 m horizontal at 0.25 m depth	1.10 m depth	1.25 m depth
Minimum RSSI reached	−116 dBm	−127 dBm	−124 dBm
Bandwidth	125 kHz	125 kHz	125 kHz
Frequency	915 MHz	868 MHz	433 MHz

**Table 10:** Comparative data with other works on different technologies.

Detail	Our Test Environment	Test Environment 1 [11]	Test Environment 2 [10]
Test environment	Pool, Yahuarcocha Lagoon, and San Pablo Lagoon	Pool	Bucket of water
Technology	LoRa	Electromagnetic (38 MHz)	Wi-Fi
Configuration	UW2OW	UW2UW	UW2UW
Depths evaluated	0.25 m, 0.5 m, and 0.8 m	2.5 m	Surface
Maximum data transfer distance	13 m (at 0.25 m depth)	5.5 m	0.15 m
Minimum RSSI Reached	−116 dBm	−80 dBm	–
Bandwidth	125 kHz	Unknown	70 MHz
Frequency	915 MHz	38 MHz	2.4 GHz

**Table 11:** Empirical operating distances for the tested UW2OW configuration.

Detail	San Pablo (SF12)	Yahuarcocha (SF12)
Maximum Distance Reached	13 m (at 0.25 m depth)	8.5 m (at 0.25 m depth)
	7 m (at 0.5 m depth)	4 m (at 0.5 m depth)
	4.5 m (at 0.8 m depth)	1.2 m (at 0.8 m depth)
Packet Loss at Max. Distance	73% (at 0.25 m depth)	98% (at 0.25 m depth)
	64% (at 0.5 m depth)	85% (at 0.5 m depth)
	70% (at 0.8 m depth)	98% (at 0.8 m depth)
Minimum RSSI at Max. Distance	−116 dBm	−116 dBm
Minimum SNR at Max. Distance	−18 dB	−23 dB
	−17 dB	−22 dB
	−18 dB	−18 dB
Empirical Operating Distance	12 m (at 0.25 m depth)	7.5 m (at 0.25 m depth)
	5.5 m (at 0.5 m depth)	3.5 m (at 0.5 m depth)
	3.5 m (at 0.8 m depth)	1 m (at 0.8 m depth)
Packet Loss at Empirical Operating Distance	45% (at 0.25 m depth)	13% (at 0.25 m depth)
	40% (at 0.5 m depth)	47% (at 0.5 m depth)
	48% (at 0.8 m depth)	30% (at 0.8 m depth)
Minimum RSSI at Empirical Operating Distance	−115 dBm	−115 dBm
Minimum SNR at Empirical Operating Distance	−16 dB	−16 dB
	−15 dB	−19 dB
	−14 dB	−19 dB

## Data Availability

The original contributions presented in this study are included in the article. Further inquiries can be directed to the corresponding author.

## Abbreviations

The following abbreviations are used in this manuscript:

LoRa	Long Range
RSSI	Received signal strength indicator
SNR	Signal-to-noise ratio
PER	Packet Error Rate
SF	Spreading factor
BW	Bandwidth
PHY	Physical Layer
NTP	Network Time Protocol
EM	Electromagnetic
dBm	Decibel-milliwatts
pH	Potential of Hydrogen
$R_{b}$	Bitrate
CR	Coding Rate
